# Design of Mucoadhesive Nanostructured Polyelectrolyte Complexes Based on Chitosan and Hypromellose Phthalate for Metronidazole Delivery Intended to the Treatment of *Helicobacter pylori* Infections

**DOI:** 10.3390/pharmaceutics12121211

**Published:** 2020-12-14

**Authors:** Maurício Palmeira Chaves de Souza, Nathalia Helena de Mattos, Liliane Neves Pedreiro, Fernanda Isadora Boni, Matheus Aparecido dos Santos Ramos, Taís Maria Bauab, Maria Palmira Daflon Gremião, Marlus Chorilli

**Affiliations:** 1 Department of Drug and Medicines, School of Pharmaceutical Sciences, Campus Araraquara, São Paulo State University (UNESP), Araraquara-SP, Road Araraquara-Jaú, Km 01, 14.800-903 São Paulo, Brazil; nathaliahelenamattos@gmail.com (N.H.d.M.); pedreiroliliane@gmail.com (L.N.P.); boni.fernanda@gmail.com (F.I.B.); 2 Department of Biological Sciences, School of Pharmaceutical Sciences, Campus Araraquara, São Paulo State University (UNESP), 14.800-903 São Paulo, Brazil; matheus.s.ramos@unesp.br (M.A.d.S.R.); tais.bauab@unesp.br (T.M.B.)

**Keywords:** mucoadhesive nanostructured polyelectrolyte complexes (nano PECs), chitosan, design of experiments, adsorption isotherms, in vitro dissolution test

## Abstract

Metronidazole (MT) is an important drug available for *Helicobacter pylori* infection treatment. However, in the past few years, this drug has presented effective reduction for infection control, one of the most important reasons is attributed to the reduction of retention time in the stomach environment. Mucoadhesive nanostructured polyelectrolyte complexes (nano PECs) based on chitosan (CS) and hypromellose phthalate (HP) were rationally developed using a full factorial design (2^1^ × 2^1^ × 3^1^), for the incorporation of MT based on the enhancement of the antimicrobial potential against active *Helicobacter pylori*, in the stomach. Different mass ratios of CS:HP (*w/w*) were tested, reaching the most promising ratios of 1:0.1, 1:0.5, and 1:1, and two methods of polymers addition (pouring-I and drip-II) were also evaluated. From method I, the obtained particles presented a diameter in the range of 811–1293 nm (Z-average) and a polydispersity index (PDI) between 0.47 and 0.88. By method II, there was a significant reduction in diameter and PDI to 553–739 nm and 0.23 at 0.34, respectively. The drug incorporation also resulted in a reduction in the diameter and PDI of the nano PECs. All samples showed positive zeta potential, about 20 mV, and a high percentage of MT incorporation (±95%). The method factor presented a greater influence on the nano PECs characteristics. Interactions in the system constituents were indicated by the FTIR data. Nano PECs mucoadhesiveness was observed and the composition and charge density were responsible for this phenomenon. MT dissolution evaluation showed the similarity of the dissolution profiles of free and loaded MT, in which almost 100% of the drug was in the simulated gastric medium in 120 min of testing. The in vitro antimicrobial potential against *H. pylori* of loaded nano PECs were measured and the minimum inhibitory concentration observed for free MT was >2000 µg/mL, while for the incorporated MT lower values were observed, showing an increase in the encapsulated MT activity.

## 1. Introduction

Infections caused by *Helicobacter pylori* infections in stomach environment are the most prevalent infection diseases all over the world, affecting around half of the world’s population [1]. Although a large part of the population present this bacteria in stomach microbiota, the presence of this microorganism can be associated with the occurrence of some gastrointestinal disorders such as gastritis, ulcers and, in some cases, gastric cancer [2].

Several therapeutic schemes based in the use of drugs with antibacterial potential has been employed for the treatment of *H. pylori* infections. One of the therapeutic strategies is the triple therapy, consisting of a proton pump inhibitor and two antibiotics: amoxicillin, clarithromycin, or metronidazole (MT) [3]. In some cases, the use of this drug association is effective to eradicate almost 91% of the bacterial load [4]. Although, the triple therapy is the most efficient therapeutic approach, the bacterial resistance to MT is more prevalent than with other antibiotics. In England, between 2000 and 2005, the MT resistance was reported in 25% of the evaluated clinical cases [5].

MT is a drug from the imidazole class, in which important therapeutic profiles are presented, such as antiparasitic, antifungal, and antibacterial [6]. This drug has a low molecular weight (171,156 g·mol^−1^) with pKa = 2.38. MT is classified as class I in the Biopharmaceutical Classification System; high solubility (in water, 11,000 mg∙L^−1^ at 25 °C) and high permeability, a limiting property for the local therapy. In addition, the high solubility and permeability associated to the oral administration, can result in systemic adverse effects, such as pain in the stomach area, nausea, vomiting, diarrhea, metallic taste in the mouth, swelling, redness, and rare but serious neurotoxicity [7,8].

The antibacterial action of MT is based on the reduction reaction, catalyzed by bacterial enzymes, in which the nitro group is reduced to a nitroanion radical, resulting in damage to the bacterial DNA. In contrast, the bacterial resistance mechanism to the MT is related to the inactivation or lesser expression of the genes responsible for the enzymes rdxA production (encodes an oxygen-insensitive NADPH nitroreductase) and frxA (encodesan NADPH flavin oxidoreductase), which are responsible for the reduction reaction [6,9]. Additionally, studies have presented that efflux pumps can act in the MT concentration reduction in the bacteria, which promotes the decrease of therapeutic efficiency [10].

In this context, the nanoencapsulation of MT into a mucoadhesive system can be an interesting alternative for MT delivery in areas colonized by bacteria, increasing the drug retention and internalization, minimizing the microbial resistance and side effects of the therapy.

Several studies show that nanostructured systems are easily internalized to improve the drug performance, due to properties related to the surface charge and the administered area [11,12,13,14,15]. Safarov and colleagues reported that the nanosized systems can specifically interact with target microorganisms through surface receptors, transporting the antibacterial agent through these structures. These authors also proposed that the particles can be adsorbed to the region and/or in the bacterial surface, releasing the drug continuously and punctually [16].

The efficiency of nanosystem based on the approaches for MT encapsulation has been continuously reported in important studies. Yeh et al. (2020) demonstrated the potential of different types of nanocarriers to convey different drugs and active substances, including MT, and the ability in the treatment of *H. pylori* infections was reported. Among the systems presented, some of those that stood out were those produced based on chitosan (CS), due to the cationic charge and, consequently, the bacterial interaction ability and the mucous membranes [17].

The mucoadhesiveness is a property that can allow the prolongation of the nanocarriers residence time in the action site, promoting a closer contact with the permeation barrier, favoring the interaction in the nano-biointerface and the local drug concentration improvement [18,19].

The use of natural or semisynthetic polymers in the development of nanocarriers is an advantageous tool due to the wide variety of structures of these materials, such as the presence of hydrophilic groups and surface charge that allow their chemical and/or physical modification, forming new materials with specific properties for a specific use [20]. The choice of polymers association, with particular characteristics such as mucoadhesiveness, enzymatic degradation resistance, and solubility affected by pH changes can result in the development of nanostructures with modulated properties, able to improve the therapeutic efficacy in diseases caused by bacterial species, especially in the treatment of *H. pylori* infections [21,22,23,24].

In this work, CS and hypromellose phtalate (HP) were selected for the nanostructured polyelectrolyte complexes (nano PECs) construction.

The CS and HP are safe, biocompatible, biodegradable, and hydrophilic polymers. CS is a polysaccharide, derived from chitin, mainly extracted from crustacean exoskeleton that, in acidified aqueous media, becomes cationic, due to the protonation of its amino groups. Its ability to establish electrostatic interactions with the mucin sialic acid residues, makes CS material with important mucoadhesive properties [25,26,27].

The HP is a cellulose derivative, widely used in the pharmaceutical industry for enteric coating films creation in solid dosage forms. Their use is able to promote a marked porosity in the systems matrix, swelling capacity and buoyancy being important parameters for gastric retention phenomena [28]. In addition, previously studies performed in our research group suggested that HP is able to be modulated by the drug methotrexate permeability and cellular accumulation, allowing the local action of the drug [29]

**Figure 1:**
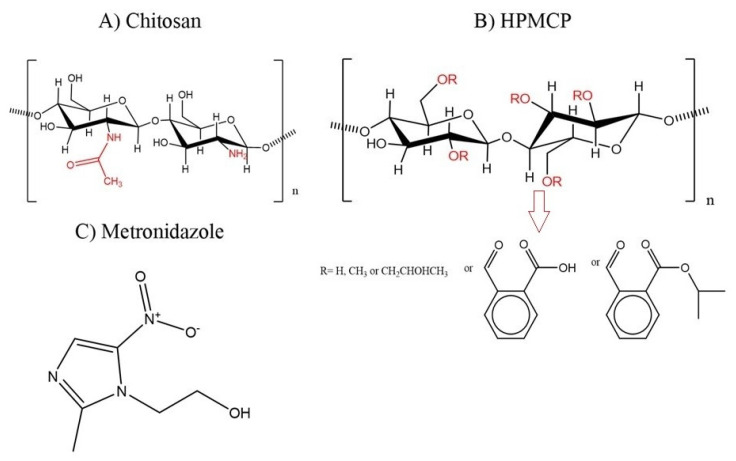
Molecular structures. (**A**) Representation of monomeric units forming chitosan, *N*-acetyl-d-glucosamine, and predominance of d-glucosamine, with acetamide and amine groups in red. (**B**) Representation of monomeric units of 2-hydroxypropylmethyl ether, phthalic acid ester (HPMCP), with substitute groups in red, indicated by the arrow. (**C**) Metronidazole.

Due to the presence of functional groups, in CS and in HP (Figure 1), it is possible to create interactions in the oppositely charged polymers to obtain supramolecular complexes. This technique is known as polyelectrolytic complexation, and has important technological advantages, such as avoiding the use of organic solvents and high energy, in addition being a relatively low cost technique [30,31]. 

Despite polyelectrolyte complexation being a technique widely used for nano PECs production, few studies explore the influence of physical-chemical parameters in the formation process. Some studies can provide valuable information in order to standardize the methodologies for nano PECs obtention, as well as the control of experimental variables, thus creating particles with desirable and reproducible characteristics. 

The aim of this study was to evaluate the process variables, using experimental design, which influences the formation of CS and HP-based nano PECs, containing MT. Nano PECs characterization and in vitro assay against *H. pylori* were performed in order to evaluate the efficacy of the developed system. 

## 2. Materials and Method

### 2.1. Materials

Low molecular weight chitosan (120 kDa; deacetylation degree of 90–95%, Sigma Aldrich^®^, St. Louis, MO, USA), hypromellose phthalate (75 kDa; phytalyl content of 31%, Shin-Etsu^®^, Tokyo, Japan), Müeller Hinton agar plus 5% sheep blood; Müeller Hinton broth supplemented with 50% fetal bovine serum; *Helicobacter pylori* bacterial strain (ATCC 4354); metronidazole (Henrifarma^®^, São Paulo, Spain); mucine type II (Sigma Aldrich^®^); resazurin (Sigma Aldrich^®^). All the other materials used were of analytical grade and obtained from commercial suppliers. Milli-Q grade water (Millipore, Molsheim, France) was used for sample preparation and the assays.

### 2.2. Methods

#### 2.2.1. Evaluation of Polymeric Ratio, MT Association, and Method Influence on the Formation and Physical-Chemical Characteristics of Nano PECs

Nano PECs were prepared by polyelectrolytic complexation method [19]. The HP dispersion (2 mg∙mL^−1^) in sodium hydroxide (0.1 mol∙L^−1^) was added into the CS dispersion (4 mg∙mL^−1^) in acetic acid (0.1 mol∙L^−1^), under magnetic stirring (Magnetic Stirrer-Fanem^®^ 258) for 60 min, at room temperature. The pH of both dispersions was adjusted to 5.5 before the obtainment process. Systems were prepared with different CS:HP ratios (*w/w*), as presented in the Table 1.

Two obtainment methods were proposed to evaluate the influence of the polymer addition in the physicochemical characteristics of the loaded nano PECs. Method I: different concentrations of MT were added in the CS dispersion and homogenized under a magnetic stirring for approximately 1 h, afterwards, the dispersion pH was adjusted to 5.5. Then, the HP dispersion was slowly dispensed over the CS-MT solution, maintaining the magnetic stirring for an additional 60 min. Method II: the HP dispersion was dripped with a syringe (0.076 mm needle) in the CS-MT dispersion, and the system was kept under magnetic stirring for 60 min. The addition of MT in different mass ratios (0.1, 0.3, and 0.5) in relation to the mass of the polymers was also tested. 

After particles selection with lower diameter and polydispersity index (PDI) and higher zeta potential, a full factorial design (2^1^ × 3^1^ × 2^1^) × 3 was employed to evaluate (quantitatively) the influence of HP proportion, MT association, and addition method on the diameter, PDI, and zeta potential of nano PECs (Minitab^®^ Statistical Software). 

The factors method and HP proportion were selected based on the parameters presented in Table 1, and were studied at two levels. The addition method factor was evaluated as categorical factors, namely as 1 and 2 for method I and method II, respectively, and HP proportions as continuous factor at 0.1 and 0.5 levels, for 0.1 and 0.5 mg∙mL^−1^, respectively. MT association at different concentrations was also studied at 0.1, 0.3, and 0.5 for 0.1, 0.3, and 0.5 mg∙mL^−1^. Twelve experiments were performed in triplicate totaling 36 assays.

#### 2.2.2. Nano PECs Characterization 

##### Diameter, PDI, and Zeta Potential Analyses

The analyses of nano PECs hydrodynamic diameter average, polydispersity index (PDI), and zeta potential were evaluated by dynamic light scattering (DLS) and electrophoretic light scattering techniques, in a Zetasizer Nano ZS^®^ equipment, at 25 °C with detection angles of 173° and 13° for size and zeta potential, respectively. Particles were analyzed in dispersion, under dilution in ultra-purified water (1:100, *v*/*v*), after production. The measurements were performed in triplicate and the results were expressed by the average of 10 measurements and the standard deviation.

These parameters of characterizations are important because they have a direct impact on the physiological behavior of the systems and the delivery profile [31,32].

##### FTIR Characterization

In order to evaluate the polymer–polymer and polymer–drug interactions, the Fourier transform infrared spectroscopy was performed for the free polymers (CS, HP) and drug (MT). For the load and free nano PECs analyses, the dispersion was previously lyophilized during 24 h (Micro Module 115, Thermo). The test was performed on a Bruker spectrometer Vertex 70 (Billerica, MA, USA) and ATR accessory, by the attenuated total reflection method (diamond crystal). For each sample 64 scans were recorded, between 4000 and 400 cm^−1^.

##### Evaluation of MT Loading Efficiency

To determine the percentage of MT loaded into the nano PECs, samples were previously frozen at −80 °C and lyophilized during 24 h. An amount of 10 mg of lyophilized nano PECs were dispersed in 10 mL of water and stirred in an Ultra-turrax mixer IKA (Staufen, Germany) for 1 min. Subsequently, samples were centrifuged at 3500 rpm for 10 min and the drug dissolved in the supernatant was quantified using a previously validated method, in a UV spectrophotometer (Agilent Technologies—Cary 60^®^) at 320 nm (y = 0.0663x + 0.0859 and r^2^ = 0.9965). 

The drug loaded was calculated according to Equation (1):

(1) %AE=(Qd/Td)×100

where %*AE* is the percentage of *MT* loaded to the system or efficiency; *Qd* is the quantified drug and *Td* is the total drug added.

#### 2.2.3. In Vitro Mucin Interaction Assay

A total of 10 mg of lyophilized nano PECs were added in 10 mL of mucin solutions in different concentrations (50, 100, 150, and 200 µg∙mL^−1^). The dispersions were homogenized by vortexing and incubated in a thermostated bath at 37 °C, for 60 min. Subsequently, the dispersions were centrifuged (Heraeus Fresco 17^®^) during 5 min at 8000 rpm. The supernatant was collected (1 mL) and 1 mL of Lowry’s reagent was added [33]. Then, 0.5 mL of the Folin–Ciocalteu’s reagent was added and the reaction was maintained for an additional 30 min under the same conditions. The absorbance was measured by visible spectrophotometry at 749 nm, in duplicate. The entire procedure was performed in a dark room.

The amount of free mucin was calculated using the analytical curve of the previously determined standard, described by the equation y = 0.0085x + 0.0741 and the determination coefficient r^2^ = 0.997. The amount of mucin adsorbed was determined from the quantification of free mucin in the supernatant, according to Equation (2).

(2) $$Q_{adsorbed\;mucin}=\;Q_{added\;mucin}\;-\;Q_{free\;mucin}$$

where $Q_{adsorbed\;mucin}$ is the amount of mucin adsorbed; $Q_{added\;mucin}$ is the amount of mucin added, and $Q_{free\;mucin}$ is the amount of mucin free.

The obtained results from the interaction of the mucin with the Nano PECs were adjusted and linearized according to the Freundlich (Equation (3)) and Langmuir (Equation (4)) equations:

(3) $$C_{ads}=KC_{e}^{1/n}$$

(4) $$C_{ads}=\;\frac{aC_{e}}{b+C_{e}}$$

where *C_ads_* is the concentration of mucin adsorbed at equilibrium (mg∙L^−1^) per unit of mass and *C_e_* is the concentration of free mucin at equilibrium (mg∙L^−1^). For the Langmuir equation, 1/*C_abs_* was plotted against 1/*C_e_* and for the Freundlich equation, the *C_abs_* log was plotted against *C_e_* to obtain the different constants (*k*, *n*, *a*, *b*).

#### 2.2.4. In Vitro Dissolution Test

The in vitro dissolution test of MT was measured using a dialysis bag and buffer HCl/NaCl (pH 1.4) media, in a Hanson SR8-Plus equipment (Chastworth, CA, USA), equipped with 150 mL vessels and apparatus I (basket). The dialysis membrane of 14,000 Da cut-off was hydrated and pretreated to remove the glycerol and other metal traces. The analyses were performed with the loaded nano PECs and the free drug. According to the sink conditions, 2 mL of MT solution (1 mg∙mL^−1^) and 2 mL of nano PECs dispersions, were introduced into the dialysis bag which was immersed in 100 mL of the media at 37 ± 0.5 °C and continuous stirring at 30 rpm. Aliquots of the media were collected at nine times intervals, during 8 h, and the same volume of fresh media was replaced. The amount of drug released was determined using the validated method (UV spectrophotometer, at 320 nm). Different mathematical models (First order, Weibull, Higuchi, Korsmeyer-Peppas, and Hixon and Crowell) were applied to the in vitro dissolution data, using the Sigma Plot 10.0 software, to evaluate what best represented the dissolution kinetics of MT and its release mechanism [34].

#### 2.2.5. Anti-*H. pylori* Activity Determination

A standard strain of *H. pylori* (ATCC 43504) was obtained from American Type Culture Collection to be used as reference strain in the activity potential determination. The minimal inhibitory concentrations (MIC) of loaded and unloaded metronidazole were measured by microdilution technique according to the protocol M100-S16 from Clinical Laboratory Standards Institute (CLSI) with modifications [35]. Initially, the microplates (96 wells) were filled with 80 μL of Mueller Hinton broth (MHB) supplemented with fetal bovine serum and 100 μL of the evaluated substances were added in the first well and 2-fold dilutions were performed to create different concentrations (free metronidazole: from 1000 to 7.8 µg·mL^−1^; HP1MT5: from 1670 to 1304 µg·mL^−1^; HP5MT5: from 1000 to 7.8 µg·mL^−1^; HP5MT3: from 600 to 4.6 µg·mL^−1^; HP5MT1: from 200 to 1.5 µg·mL^−1^) and 20 μL *H. pylori* suspension at 6 × 10^7^ CFU·mL^−1^ were deposited in each well. The microplates were incubated at 37 °C during 72 h in an incubator with 10% of CO_2_ and humidity.

At the end of the incubation period the MIC values were measured by addition of 30 μL of a resazurin solution (100 µg·mL^−1^) followed by 2 h of incubation in the same incubation parameters as described previously in which the presence of blue color represents the absence of bacterial growth and of pink color, the presence of bacterial growth [36]

The control of the culture medium, bacterial growth, positive inhibition control with amoxicillin, sterility samples controls, and negative control (solvent and free nanostructured systems) were also performed.

#### 2.2.6. Statistical Analysis

The results were treated by one-way analysis of variance to assess the significance of the differences between data. Tukey–Kramer post-hoc test was used to compare the means of different treatment data (Origin 7.0 software). Results with *p* < 0.05 were considered statistically significant. A full factorial design (2^1^ × 3^1^ × 2^1^) × 3 was applied to the data (Minitab^®^ Statistical Software).

## 3. Results and Discussion

### 3.1. Evaluation of the Polymeric Ratio and Method on the Nano PECs Obtention

The samples prepared by method I, HP 12.5 and HP 15, in which the mass of HP was higher than the CS mass (Table 1, Section 2.2.1), the formation of large aggregates and phase separation was observed. Possibly, the higher proportion of HP results in the reduction of particles zeta potential, due to the charges annulment from the protonated amine groups (*NH*_3_*^+^*) of CS and the carboxylate group (*COO*^–^) of HP. This reduction in the zeta potential, near to zero, should result in a reduction in the electrostatic repulsion of the particles and, consequently, in their attraction and aggregation [37].

Samples HP 1, HP 5, and HP 10 showed an opalescent visual aspect [19]. This opalescent aspect is the result of the Tyndall effect, due to the interference in the light passage through the system. The observation of the Tyndall effect in the systems indicates the nanostructured particles formation [38]. Samples HP 1, HP 5, and HP 10 were selected for characterization according Section 2.2.2 and the results are shown in Table 2.

The nano PECs diameter ranged from 734 to 810.8 nm. The diameter is one characteristic responsible for the cellular uptake, smaller particles tend to be more easily internalized by cells [39].

Nano PECs showed positive surface charge between +17.5 and +25.7 mV (Table 2), it means that the zeta potential of nano PECs is more strongly influenced by CS. Modular zeta potential values around 25–30 mV are desirable for the physical stability of the system, due the interparticle electrostatic repulsion [31,32]. The positive charge is also interesting to favor the interaction with the anionic group of lipids of the cell membrane or to the mucous, formed by monosaccharide sialic acid [40].

By Table 2, it was also possible to observe that with the increase in the HP ratio from 0.1 to 1, the zeta potential of the nano PECs decreases from +25.7 to +17.5 mV.

This reduction in zeta potential may occur because the HP molecules preferably associate more closely in the external region of the particle, due to their more rigid and bulk chain. The HP chains must have difficulty interpenetrating the formed precomplex, remaining more in the external region, giving a less positive character to nano PECs [41,42].

The analysis of PDI shows values in the range of 0.60–0.88. PDI represents the deviance of the particles size distribution, PDI < 0.4 indicates that the particles present low defiance in size [43].

According Table 2, nano PECs obtained by higher proportions of HP presented higher PDI values and consequently, the size distribution is less homogeneous.

This may be related to the polymeric dilution regime in the medium. With more polymer chains competing for solvent in the medium (concentrated regime), the probability of thermodynamically oriented arrangements are less, the kinetically oriented arrangements prevailing due to the reduction mobility in the medium, as well as the conformational limitations of the chains in unfolding [44].

Nano PECs were formed in regions with high polymer concentrations (center of the container) to have larger polymer clusters and regions with lower concentrations of these polymer chains (periphery of the container). In these regions with lower polymeric density there is greater configurational and conformational freedom, for more uniform and thermodynamically oriented arrangements of the polymer chains.

Following the studies, the load of MT and the complexes obtainment was performed according to the two methodologies mentioned in Section 2.2.1, and the characteristics of these nano PECs as presented in Table 3.

According to Table 3, the association of MT resulted in an increase in the average diameter of nano PECs, compared to the empty nano PECs, with some samples reaching the micrometric size (>1000 nm). Probably, when MT was added, it interacted with the polymers in order to decrease the probability of CS-HP interactions, which is an organized association, forming a more disordered matrix, with higher dimension.

Results in Table 3 also showed that for HP 1 and HP 5 the drug association resulted in a reduction in the PDI value from 0.60 and 0.78 to values between 0.47 and 0.52. This effect was not observed for sample HP 10, with a 1:1 ratio of CS: HP, which even after the drug association the higher PDI values were maintained (>0.80). For samples composed by higher polymeric proportions (1:1) the polymer–polymer interactions are favored due to the high probability of the polymer chains being found, becoming less sensitive to the influence of MT addition. In conditions where the likelihood of encounters between polymer chains is low, interactions between MT–polymer are favored, in order to alter the formation of nano PECs [45].

As the HP10 sample showed a high PDI value and decreased zeta potential, which may be indicative of low physical stability, it was discarded from the test of obtainment by method II (dripp).

Seeking better results in diameter and PDI, the nano PECs were obtained by dripping (method II).

The results of nano PECs obtained by method II are shown in Table 4. The gradual and slow addition of the polymer allows the conditions of thermodynamic equilibrium to be established to a greater extent, as in this case the system has greater conformational and configurational freedom, which should result in more intense interactions between CS and HP, resulting in smaller and homogeneous particles [44].

For all the samples analyzed, practically all the mass of MT added in the system was incorporated into the structure of the nano PECs and the association efficiencies were greater than 95%. Through the analysis of the content of drug associated, it was possible to infer the efficiency of the developed system. From the results obtained, nano PECs are classified as systems with high capacity for drug incorporation, showing itself as a promising system in the MT delivery. Regardless of the HP and MT amount, the selected nano PECs had a high association capacity.

From the data obtained in the experiments, method I proved not to be suitable for obtaining particles on a nanometric scale with homogeneous size distribution.

Among the nano PECs obtained by method II, the HP 1 and HP 5 systems presented the most suitable characteristics for the application, such as reduced diameter, low PDI, and high ZP values.

Then the values of polymeric ratios and drug were used together with the mixing method to compose a full factorial experiment (2^1^ × 3^1^ × 2^1^) as shown in Table 5, with the aim to assess the influence and effect of the interaction of these factors on the size, PDI, and ZP of the nano PECs.

**Figure 2:**
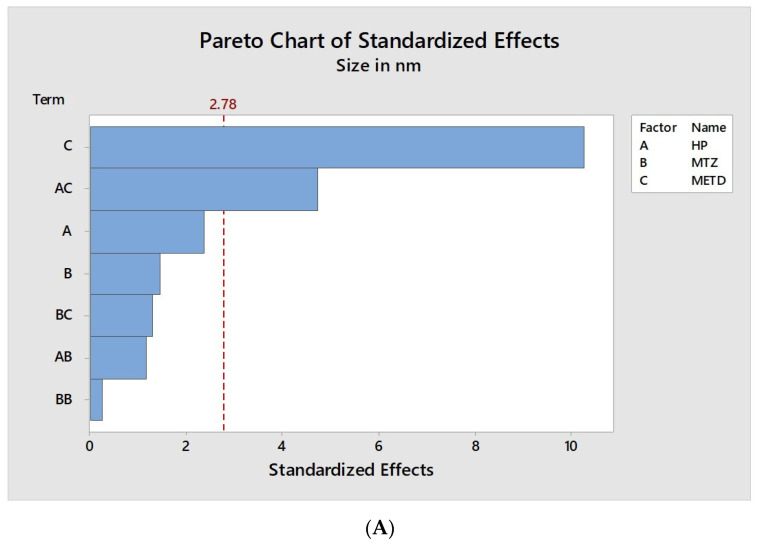

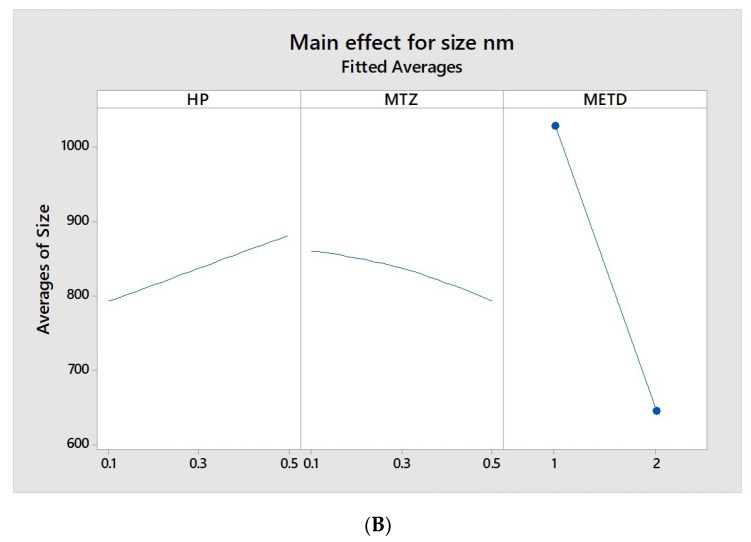
Graphic representation of factor influences on average size of particles. (**A**) Standardized Pareto chart estimating the effect of mass ratio HP, mass ratio MT, method, and their interaction on the size of the particles. The red dashed vertical line represents the 0.05 critical value for ANOVA. All bars extending to the right of this line indicate that the effects are statistically significant at 5% significance level. (**B**) Isolated analysis of the factors, HP/MT concentration, and method (on *X*-axis), and their influence on the size of the nanoparticles (on *Y*-axis).

**Figure 3:**
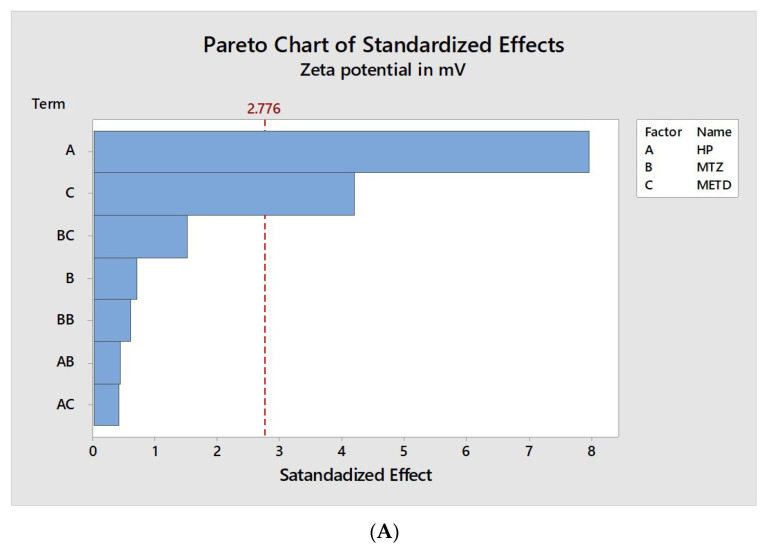

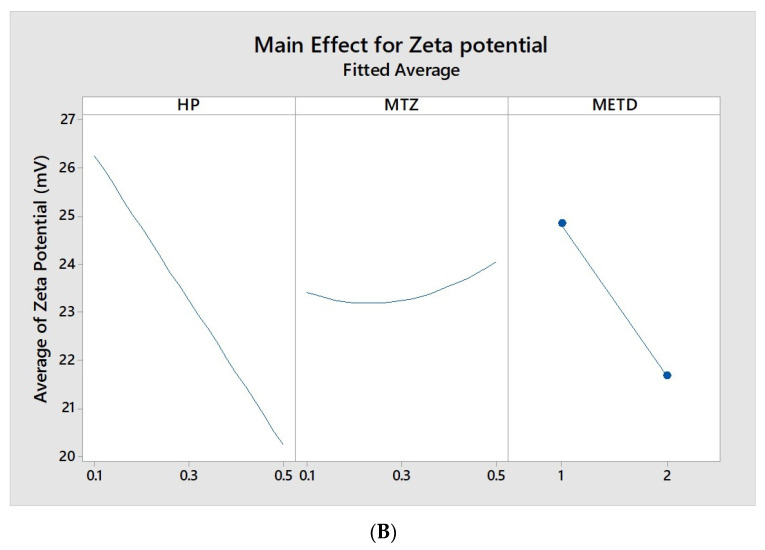
Graphic representation of factor influences on average PDI of particles. (**A**) Standardized Pareto chart estimating the effect of mass ratio HP, mass ratio MT, method, and their interaction on the PDI of the particles. The red dashed vertical line represents the 0.05 critical value for ANOVA. All bars extending to the right of this line indicate that the effects are statistically significant at 5% significance level. (**B**) Isolated analysis of the factors, HP/MT concentration, and method (on *X*-axis), and their influence on the PDI of the nanoparticles (on *Y*-axis).

**Figure 4:**
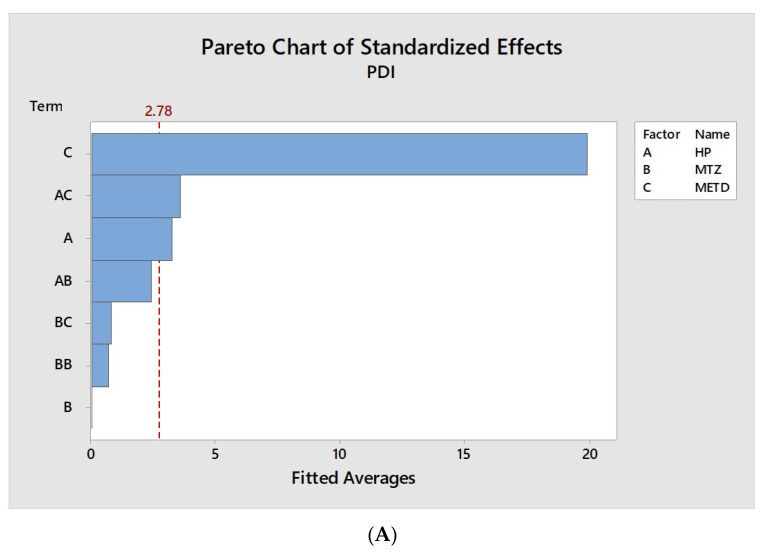

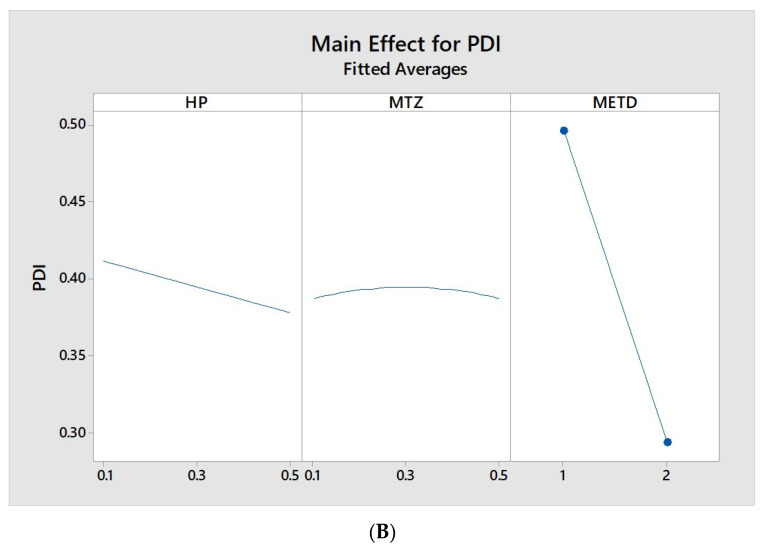
Graphic representation of factor influences on average zeta potential of particles. (**A**) Standardized Pareto chart estimating the effect of mass ratio HP, mass ratio MT, method, and their interaction on the zeta potential of the particles. The red dashed vertical line represents the 0.05 critical value for ANOVA. All bars extending to the right of this line indicate that the effects are statistically significant at 5% significance level. (**B**) Isolated analysis of the factors, HP/MT concentration, and method (on *X*-axis), and their influence on the PDI of the nanoparticles (on *Y*-axis).

The analysis of each factor’s influence and their interactions with the nano PECs characteristics are shown in Figure 2, Figure 3 and Figure 4. They contain a bar for each effect, sorted from most significant to least significant. The length of each bar is proportional to the standardized effect. A vertical line is drawn at the location of the 0.05 critical value for the statistical test.

The results observed in Table 4 together with the data in Figure 2A, reinforce the fact that the average diameter of the nano PECs is mainly dependent on the method and the interaction between the method and the HP proportion. In Figure 2B it is possible to verify the strong influence of the obtainment method on the average diameter of the nano PECs. While method I formed particles with average sizes varying between 871 and 1292 nm, method II (drip) formed particles with diameters between 553 and 664 nm, concluding that quantitatively method II is the most suitable for the formation of smaller nano PECs.

In Figure 3, is possible to observe the influence of method, HP ratio, and MT in the PDI of nano PECs. A similar result to that of diameter, the method strongly influenced the PDI of nano PECs, in which the average values obtained by method I were ±0.49 while the average observed by method II was ±0.26, showing a homogeneity of particle diameter distribution, when it was obtained by dripping. They indicate that the method II was more efficient in reducing the average particle diameter, as well as, the PDI.

Finally, the influence of the factors tested on the zeta potential of the nano PECs was evaluated and the results showed in the Figure 4, once again, a significant influence of the method and HP proportion were observed. As was discussed previously, the HP contributes to the lower positive charge density of nano PECs, due to it anionic nature and its conformation during the nano PECs formation.

A possible explanation for the differences in zeta potential, observed between the nano PECs obtained by the different methods, may be the fact that in method I, large amounts of HP were added to the reaction medium, containing QS-MT, this favored the formation of larger particles, as there was no sufficient time for the complete dispersion of HP in this medium and the electrostatic interactions, Van der Waals forces, and hydrogen bonds between the nearby chains, of both polymers, were quickly established, forming particles containing polymeric tangles, with discontinuous and interstice regions containing MT. Thus, it can be considered that the formation process of these nano PECs by method I was governed kinetically. As it is a dynamic and kinetically controlled process, the lack of control when adding the HP dispersion, would promote the formation of nano PECs with varying diameters, impacting in the PDI [44].

In the systems obtained by drip method II, in which the addition of HP was carried out in a controlled and slow manner, the process would be governed thermodynamically, since smaller amounts of HP were introduced in the medium, allowing this material to disperse and interact with the nearby CS chains, slowly and initially due to strong, electrostatic interactions. As these interactions occurred, the loads of these complexes would become smaller and CS chains with higher loads would interact with the new available HP chains, dripped in the medium, so that the formation of these complexes would be associated with the reduction of the system’s overall energy by reducing polymer loads. These complexes would present smaller PDI values and smaller particle diameters as well, since in these cases the interactions would be much more governed by electrostatic interactions than other types of interactions, a fact evidenced by the low values of zeta potential (Table 3 and Table 4). Based on all the results and discussion, method II was selected to obtain the nano PECs. Thus, all samples analyzed in the subsequent tests were obtained using this method.

### 3.2. Fourier-Transform Infrared Spectroscopy (FTIR)

**Figure 5:**
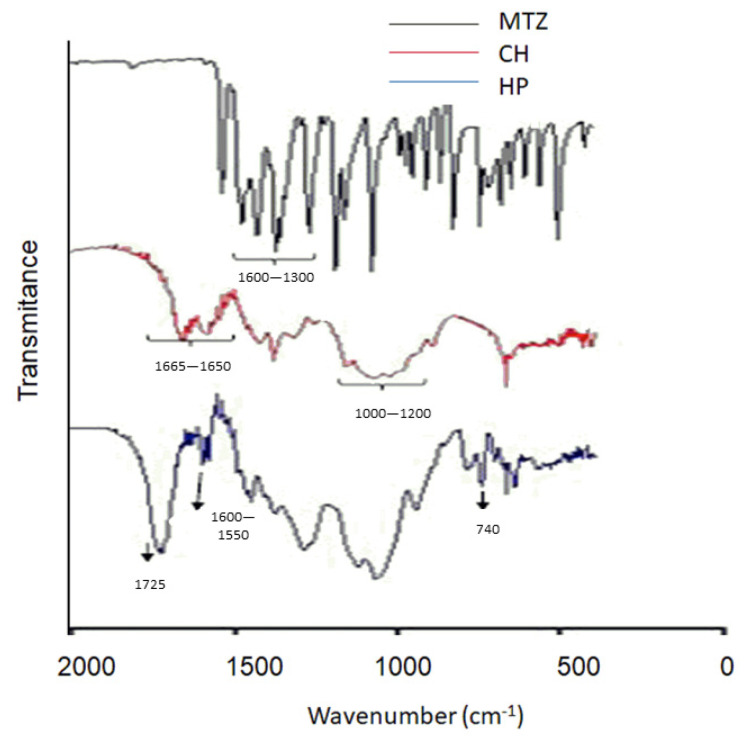

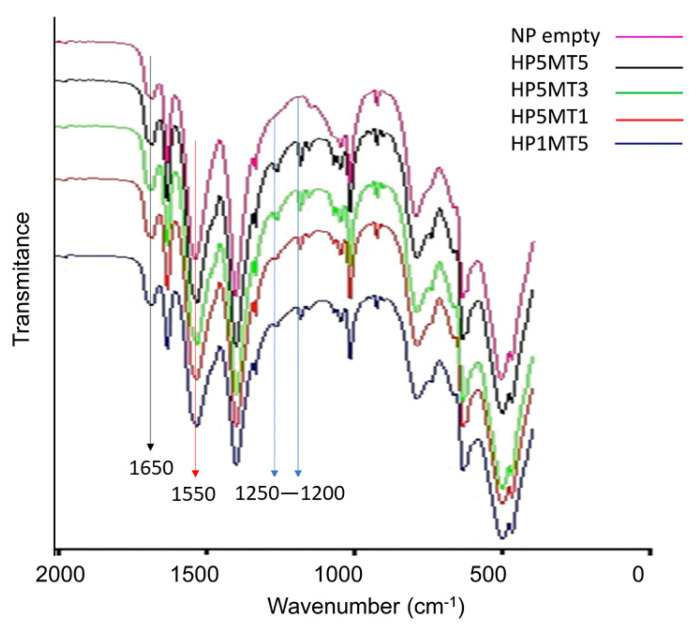
(**A**) Infrared spectra of MT and polymers. (**B**) Infrared spectra of nano PECs of groups HP 1 and HP 5 and empty NP.

Figure 5A shows the absorption spectra of CS, HP, and MT in the infrared region. In the MT spectrum, bands in the regions from 1300 to 1600 cm^−1^ are assigned to the *NO*_2_ group. The bands at 2900 and 3100 cm^−1^ represent stretching of sp^3^ and sp^2^ carbons, respectively. The bands in the regions of 3200–3600 cm^−1^ are attributed to the stretch of the *O–H* group [46].

In the CS absorption spectrum, bands were observed in the regions from 1650 to 1665 cm^−1^, which can be attributed to the *C=O* stretch from the secondary amide group. The bands from 1560 to 1610 cm^−1^ are attributed to the axial deformation of the *NH_2_* group in the plane, the bands from 1200 to 1000 cm^−1^ are attributed to the *CO* stretching, and the bands from 3350 to 3180 cm^−1^ to the stretching of the NH_2_ group [47]. In the HP absorption spectrum, bands from 3500 to 3200 cm^−1^ were noted, attributed to the stretching of the *O–H* group. The band observed at 2800 cm^−1^ is attributed to the methoxy group (*C–CH*_3_) and at 1725 cm^−1^ it is attributed to the stretching of the *C=O* group of the ester group; from 1600 to 1550 cm^−1^ attributed to the aromatic ring and 740 cm^−1^ attributed to the monosubstituted aromatic ring [48].

Figure 5B shows the absorption spectra of empty and drug-containing nano PECs. It is possible to notice in the loaded nano PECs the presence of the characteristic band of the MT between 1200 and 1400 cm^−1^, which is not noticed in the empty samples. Between 3500 and 3100 cm^−1^ referring to *NH_2_* and *OH*, it can be noted that there was a displacement of the bands in all samples, when compared to CS, HP, and MT, which may indicate the interaction of protonated amino groups of CS with the carboxylic groups HP, as well as the MT NO and *OH* groups of CS and the *C=O, C–O*, and *OH* groups in HP through dipole–dipole interactions and hydrogen bonds. In addition, the differences found in the 500–1700 cm^−1^ bands of the samples, in relation to the free polymers, can also suggest an interaction between them.

It is possible to see two bands, one at 1200 and the other at 1250, only in systems with MT. These bands are related to stretching of the C-N bonds, abundant in MT. One observation that confirms this statement is the fact that both bands are becoming less intense as the concentration of MT in the systems is reduced [49]. At 1650 cm it is possible to identify an intense band in loaded nano PECs spectra, which is low in the empty nano PECs spectrum. This increase in the intensity of the C=C carbonyl band, already present due to the double bonds of the aromatic rings of the HP, may be related to the presence of a C=C bond in the imidazolidinic ring [49,50]. At 1550 cm^−1^, related to NH_2_ stretch, abundant in CS, it is possible to observe the reduction in the intensity of this band, which is quite intense in the empty nano PECs, and becomes progressively less intense, as the incorporations of MT occur. This observation may indicate that there are interactions between the NH_2_→NH_3_^+^ groups of chitosan with the NO^−^ groups present in metronidazole [51]. In Table 3, it is possible to see that the addition of MT in the nano PECs leads to a reduction in the system’s PDI. The reduction in the PDI value can be associated with the fact that MT may be interacting with CS, forming more monodispersed systems. The effect of adding MT on the systems stems from the fact that MT can interact more with polymers, especially with CS, as evidenced in the FTIR spectrum, forming larger particles, however, despite the particles having a larger diameter, with a greater probability of interaction, we will have more uniformly distributed particles [52].

### 3.3. In Vitro Mucin Interaction Assay

**Figure 6:**
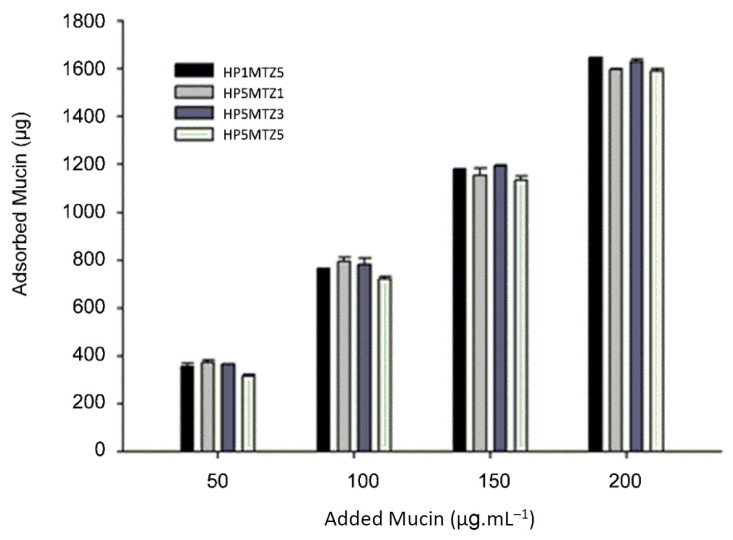
Amount of mucin adsorbed by nano PECs according to the different masses of mucin added (±SD).

Figure 6 shows the results of mucin adsorption on nano PECs. It was observed that for all samples the amount of adsorbed mucin increased with the increase in the amount of added mucin, highlighting the high mucoadhesiveness of the nano PECs.

The nano PECs mucoadhesion derives from the fact that CS, predominant in nano PECs composition, in acidic media, such as the stomach, present protonated amino groups. Mucus is mainly composed by mucin (pKa-2,6), a glycoprotein and its sialic acid residues, in acid pH values, are ionized presenting negative charge. Electrostatic interactions and Van der Waals forces must be established between the system surface and the mucin, favoring the adsorption [52,53].

To the mucin adsorption data, Freundlich and Langmuir models were applied and the results are shown in Table 6.

The Freundlich model describes the adsorption of a single solute, on the adsorbent, in multiple layers and in a reversible way, assuming that the solid surface is irregular and heterogeneous [54]. Langmuir’s model considers the adsorption on a homogeneous surface, in monolayer, over the entire surface of the adsorbent, in which the adjacent molecules of the solute do not interact with each other, remaining in solution [20].

For HP1MT5 the adsorption was better adjusted to the Langmuir model, this particle presented a greater positive zeta potential. Therefore, the adsorption must be governed by electrostatic interactions, between its surface and mucin, which adsorb quickly due to electrostatic attraction. These interactions are strong and are established at shorter distances, must be able to form a homogeneous mucin layer, and closely adhered to the particle surface [55]. In this case, the system has better adhesivity and more force of adhesion to the negative surfaces, like cells and mucous layers. We believe that mucin adsorbed in the surface of positive nano PECss it actuate as a repulsive layer, not leaving other nano PECs to approximate the negative mucin negative and adsorb to that, forming multiple absorption layers (particle-mucin) [56].

The other systems listed in Table 6 are more fitted to the Freundlich model. A possible explanation for this is the fact that these systems also have positive zeta potential, although, relatively lower <+20 mV. Possibly, the interactions that occur between mucin and these system surfaces also are majority electrostatic type, however, some weaker interactions such as hydrogen bonds and Van der Waals forces, are established at a greater distance than when compared to the HP1MT5 system, making this type of adsorption less intense [56].

In addition, the lower zeta potential allows interactions between nano PECs, making it possible to establish adsorption in multilayers, that is, nano PECs already with mucin adsorbed on its surface can interact with other nano PECs, increasing the number of layers on the adsorbent surface [57].

Regarding the *n* coefficient, it indicates the intensity of mucin adsorption with the surface of the particles, *n* values greater than 1.0 represent favorable conditions for adsorption [58,59]. Thus, all particles, even with higher concentrations of MT, showed strong adsorption interaction. The *k* coefficient is related to the adsorption capacity. For HP1MT5, with a lower ratio of HP and HP5MT5 with a higher concentration of MT, the values found were lower, which may indicate that the addition of drug reduces the adsorption capacity, perhaps due to the formation of a more compact matrix and less positive residual load, which makes it difficult to interact with mucin molecules [60].

### 3.4. In Vitro Dissolution Test

Based on the data found for the adsorption isotherms and zeta potential values, samples HP1MT5 and HP5MT3 were chosen to perform the in vitro dissolution test. These systems were also chosen because they have higher and lower zeta potential values, respectively, and variations on the release profile can be justified by some of these physical-chemical characteristics. Free MT was also tested, to evaluate the influence of nano-compartmentalization on the drug dissolution rates.

**Figure 7:**
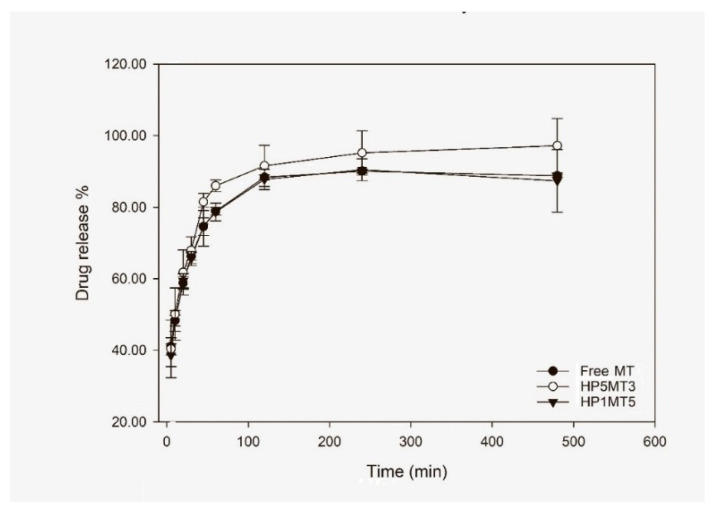
Graphical representation of the means and standard deviations of the results of the release test.

The results in Figure 7 suggest that both the free MT and the MT incorporated in the HP1MT5 system present statistically similar dissolution profile and release rates.

Around 100% of the MT was released at 120 min (Figure 7). This may be associated with the fact that in strongly acidic media (stomach pH = 1.5–3.5), CS quickly becomes protonated. The extensively protonated CS chains electrostatically repel, consequently, the matrix swelling allowed the diffusion of medium to the particle structure, dissolving the drug and favoring the diffusion to the dissolution medium. In addition, the proton-rich solvent accesses the HP chains by neutralizing their charges, this event can culminate in the disruption of the particle matrix, also favoring the rapid release of the drug.

The best model that represented the dissolution kinetics of MT was the Korsmeyer–Peppas (Equation (5)).

(5) F=(Mt/M)=k×tn

where *F* is the total drug concentration in the medium, *Mt* is amount of released drug in at time “*t*”, *M* is amount total drug in the system *k* is the constant of incorporation of structural modifications and geometrical characteristics of the system (also considered the release velocity constant), and *n* is the exponent of release (related to the drug release mechanism) as a function of time t [61,62].

In all systems, a value of *n* greater than 0.5 and lower then 1.0 (Table 7) was observed, such experimental data indicate that the observed release profile can be classified as anomalous, in which the drug release was controlled by a combination of many processes, including the swelling and dissolution of the polymeric matrix, diffusion and drug dissolution, as discussed above [63].

The results indicate that the system did not allow a prolonged release of the drug. However, considering the high mucoadhesive capacity of the developed system, in the physiological environment a large number of nano PECs can adhere to the mucous layer, resisting gastric emptying, since due to the small diameter the nano PECs behave similarly to liquids and may remain in the stomach for less time, resulting also in a close contact with *H. pylori* [64]. In addition, with the nano PECs matrix erosion, the polysaccharides can interact with the bacteria membrane, resulting in destabilization and, consequently, in the greatest internalization of the drug [64] and/or acting in the inhibition of membrane efflux pumps.

### 3.5. Anti-H. pylori Activity

The results of the antibacterial activity of free metronidazole and loaded into nanostructured systems are presented in Table 8.

The MIC found for the formulation that showed antibacterial activity (HP1MT5) was 835 µg∙mL^−1^. This sample, compared to the others analyzed, contained a higher concentration of MT in relation to HP, a factor that can substantiate the result obtained. Another important aspect to be taken into account is related to the data obtained for the mucoadhesion mechanism, in which the HP1MT5 system is better adjusted to the Langmuir isotherm, a phenomenon attributed to its high zeta potential. As it is able to establish stronger electrostatic interactions, compared to the other systems, these nano PECs are likely to interact with the bacterial surface forming a monolayer adsorbed to the cell, allowing the drug to be internalized more efficiently [65]. Another sample that demonstrated an interesting result was HP5MT5

## 4. Conclusions

Nano PECs based on CS and HP were successfully obtained by the polyelectrolytic complexation technique. By the results, HP proportion and the obtaining method were the parameters that directly influenced the characteristics of the nano PECs. Lower ratios of HP and the slow polymer addition by dripping (method II), demonstrated to be the most appropriate conditions for obtaining particles with diameters smaller than 700 nm, PDI lower than 0.4, and zeta potential higher than 19 mV. The nano PECs showed a high capacity for incorporating MT, but no ability to control the drug release rates in an acidic medium was observed. However, the nano PECs mucoadhesiveness was proven by mucin adsorption assay, and this property can be favorable to the system overcoming the challenge of rapid drug release. HP1MT5 system was shown to be the most promising in relation to in vitro anti-*Helicobacter pylori* activity, presenting antibacterial performance, when compared with the other systems and free MT. Thus, the incorporation of MT in mucoadhesive delivery systems presents a promising system for the treatment of gastric infections caused by *H. pylori*. Based on this information, in vivo studies should be conducted seeking to deepen the study of the biological behavior of the developed system.

## Figures and Tables

**Table 1:** Code and composition of the systems.

Sample	Polymer Ratio (CH:HP *w/w*)
HP 1	1:0.1
HP 2	1:0.2
HP 3	1:0.3
HP 4	1:0.4
HP 5	1:0.5
HP 5.5	1:0.55
HP 7.5	1:0.75
HP 10	1:1
HP 12.5	1:1.25
HP 15	1:1.5

**Table 2:** Particle diameter, zeta potential, and polydispersity index (PDI) as a function of the CS:HP ratio.

Sample	Diameter Z-Average (nm)	PDI	Zeta Potential (mV)
HP 1	795.4 ±12.8	0.60 ± 0.01	25.7 ± 1.1
HP 5	810.8 ± 108.8	0.78 ± 0.19	21.1 ± 1.4
HP 10	734.0 ± 70.9	0.88 ± 0.10	17.5 ± 0.9

**Table 3:** Particle size, zeta potential, and PDI of nanoparticles containing MT prepared by method I.

Sample	Mass Ratio (CS:HP:MT)	Diameter Z-Average (nm)	PDI	Zeta Potential (mV)
HP 1	1:0.1:0	795.4 ± 12.8	0.60 ± 0.01	25.7 ± 1.1
HP1MT1	1:0.1:0.1	916.3 ± 50.1	0.47 ± 0.07	29.7 ± 1.7
HP1MT3	1:0.1:0.3	878.8 ± 115.9	0.49 ± 0.07	27.2 ± 1.4
HP1MT5	1:0.1:0.5	871.1 ± 59.7	0.51 ± 0.02	28.0 ± 0.9
HP 5	1:0.5:0	810.8 ± 108.8	0.78 ± 0.19	21.1 ± 1.4
HP5MT1	1:0.5:0.1	1292.7 ± 155.8	0.49 ± 0.08	22.2 ± 0.9
HP5MT3	1:0.5:0.3	1087.0 ± 22.3	0.52 ± 0.09	21.4 ± 1.1
HP5MT5	1:0.5:0.5	1085.3 ± 9.6	0.47 ± 0.05	22.4 ± 1.4
HP 10	1:1:0	734.0 ± 70.9	0.88 ± 0.10	17.5 ± 0.9
HP10MT1	1:1:0.1	927.8 ± 83.4	0.77 ± 0.03	16.9 ± 0.3
HP10MT3	1:1:0.3	981.6 ± 167.2	0.87 ± 0.05	16.6 ± 0.4
HP10MT5	1:1:0.5	953.2 ± 126.9	0.77 ± 0.04	16.8 ± 0.4

**Table 4:** Particle size, PDI, and zeta potential of loaded nano PECs prepared by method II (drip) as a function of HP and MT proportions.

Sample	Mass Ratio (CS:HP:MT)	Diameter Z-Average (nm)	PDI	Zeta Potential (mV)	EA (%)
HP1MT1-2	1:0.1:0.1	644.8 ± 83.1	0.32 ± 0.06	22.5 ± 0.7	99.7
HP1MT3-2	1:0.1:0.3	739.1 ± 38.6	0.31 ± 0.01	25.7 ± 0.7	99.6
HP1MT5-2	1:0.1:0.5	664.0 ± 28.0	0.34 ± 0.07	26.3 ± 1.9	99.5
HP5MT1-2	1:0.5:0.1	586.8 ± 10.7	0.27 ± 0.03	19.2 ± 0.3	99.7
HP5MT3-2	1:0.5:0.3	644.2 ± 12.2	0.26 ± 0.01	18.7 ± 0.2	99.8
HP5MT5-2	1:0.5:0.5	553.0 ± 26.3	0.23 ± 0.01	19.5 ± 0.9	99.6

**Table 5:** Design of experiments with codified factor levels for the full factorial design.

Method I	Codified Level	Method II	Codified Level
HP1MT1-I	−1:−1:−1	HP1MT1-II	−1:−1:1
HP1MT3-I	−1:0:1	HP1MT3-II	−1:0:1
HP1MT5-I	−1:1:−1	HP1MT5-II	−1:1:1
HP5MT1-I	1:−1:−1	HP5MT1-II	1:−1:1
HP5MT3-I	1:0:−1	HP5MT3-II	1:0:1
HP5MT5-I	1:1:−1	HP5MT5-II	1:1:1

**Table 6:** Constants obtained by linearizing the Freundlich and Langmuir equations.

Sample	Zeta Potential		Freundlich Isotherm (a)			Langmuir Isotherm (b)		
			*k*	1/*n*	*r* ^2^	*A*	*b*	*r* ^2^
HP1MT5	26.3	0.4928	0.6221	0.9864	0.0082	0.5101	0.9972	
HP5MT1	19.2	2.0687	0.8572	0.991	0.0031	0.3662	0.9704	
HP5MT3	18.7	0.8894	0.6937	0.9983	0.0063	0.4405	0.993	
HP5MT5	19.5	0.1086	0.5124	0.9953	0.0143	0.8343	0.9918	
	logQe=logk+1n x log Ce		(a)					
	1Ce=a+b x 1Ce		(b)					

**Table 7:** Constants of Korsmeyer–Peppas adjusted model.

System	*k*	*n*	*R*	*R* ^2^
HP1MT5	0.0039	0.5930	0.9286	0.8623
HP5MT3	0.0075	0.5628	0.9187	0.8439

**Table 8:** Antibacterial activity of free metronidazole and loaded into nanostructured systems against. *H. pylori* ATCC 43504.

Sample	Max Analyzed Concentration	MIC Values *
MT	2000	>2000
HP1MT5	1670	835
HP5MT5	1000	>1000
HP5MT3	600	>600
HP5MT1	200	>200
Amoxicillin	2000	0.007
Free nanostructured systems	-	-

* Values expressed in µg∙mL^−1^; (-): no interference; MTZ: free metronidazole; HP1MTZ5, HP5MTZ5, HP5MTZ3, and HP5MTZ1: metronidazole loaded into nanostructured systems.

## Abbreviation

AE	Association efficiency
ATCC	American Type Culture Collection
ATR	*FTIR*-attenuated total reflectance Fourier transform infrared
CS	Chitosan
*H. pylori*	*Helicobacter pylori*
HP	Hypromellose phthalate
METD	Method
MHB	Mueller Hinton broth
MT	Metronidazole
Nano PECs (NP)	Nanostructured polyelectrolyte complexes
PDI	Polydispersity index
ZP	Zeta potential
